# Transtheoretical model-based nutritional interventions in adolescents: a systematic review

**DOI:** 10.1186/s12889-020-09643-z

**Published:** 2020-10-14

**Authors:** Jennifer Nakabayashi, Giselle Rha-isa Melo, Natacha Toral

**Affiliations:** Department of Nutrition, University of Brasilia, Center for Epidemiological Health and Nutrition Studies, Brasilia, Distrito Federal Brazil

**Keywords:** Adolescent, Dietary intake, Transtheoretical model

## Abstract

**Background:**

Literature has shown a tendency of inadequate dietary intake among youth, consequently, nutritional interventions are required. The transtheoretical model (TTM) classifies individuals based on their readiness to change. This model is widely used for health education interventions with proven efficacy.

**Purpose:**

This review aimed to weigh the strength of evidence about the TTM usage in nutritional interventions for adolescents and its effectiveness regarding dietary intake.

**Methods:**

This study followed the PRISMA guidelines. Eligible studies were input into Mendeley software. The Adolec, Google Scholar, LILACS, PsycINFO, PubMed, Science Direct and Web of Science databases were searched. Only full original articles written in English, Spanish or Portuguese on randomized controlled trials and quasi-experimental designs that applied the TTM in the design of nutritional interventions targeting adolescents were included, with no restrictions on publication date. The quality and risk of bias was evaluated with the Effective Public Health Practice Project Quality Assessment Tool for Quantitative Studies.

**Results:**

The initial search yielded 3779 results. Three studies were rated as strong, six as moderate and five as weak. The final sample of 14 articles included adolescents that were mostly recruited from schools, with interventions ranging from one month to three years. The TTM was used alone or combined with other behavior-change theories and most of the interventions involved digital technology. The nutritional topics covered included fruit and vegetable consumption, low-fat diet, and cooking skills. Four studies presented improvement in fruit and vegetable consumption and four progressed through stages of change. Participants from two interventions reduced fat intake. At the end of one intervention, all the participants were in action and maintenance stages.

**Conclusion:**

The TTM seems to be a successful strategy for nutritional intervention aiming at improving dietary intake in adolescents. Its application in different contexts shows that the TTM is flexible and possible to be implemented in many settings. The use of the model is shown to be restricted to the stage of change’ construct. Further studies should use all constructs of the TTM in the design and compare the TTM with other behavior-change theories to better understand its effectiveness.

## Background

Adolescence is a period of intense biopsychosocial changes and involves specific nutritional needs [1, 2]. Healthy eating habits are essential to appropriate growth and development in this age group and serve as a protective factor against non-communicable diseases [1, 2]. A tendency toward inadequate eating habits among youth has been documented in the literature, including high intake of sugary drinks [3], skipping breakfast [4] and low consumption of fruits and vegetables [5]. Thus, nutritional intervention is required to ensure healthy growth and prevent the development of chronic diseases while still young [6].

The transtheoretical model (TTM) describes change not as an individual event but rather as a series of steps that take place according to a person’s degree of motivation. The four constructs of TTM are the stages of change, the processes of change, self-efficacy, and decisional balance [7]. The most explored construct is the five stages of change, that have been determined based on motivation level. Individuals in the precontemplation stage are unaware that their behavior is harmful and are not well informed about how to change, so their motivation is low or nonexistent [7]. Individuals in the contemplation stage are less reluctant to change and they may be aware that there is something wrong with their behavior but are unwilling to act. Individuals in the decision stage feel more prepared to act and plan to change according to short-term goals. In the action stage meaningful changes in behavior occur, while in the final stage, maintenance, the new behavior persists for at least six months [7].

Different processes are involved in progression through the stages of change. For example, to cross from precontemplation into contemplation, consciousness raising must occur, and this process mostly applies to initial stages. As individuals progress through the stages, their self-efficacy is expected to increase, i.e. how capable they feel of changing, including recognition that there are more pros than cons to changing, as stated in decisional balance [7].

This model is widely used for health education interventions, such as physical activity [8], fruit and vegetable consumption [9] and weight control [10], with proven efficacy. Some positive aspects of using the TTM include its low-cost, its adaptability to several problems [8], and the fact that the intervention can be tailored for participants according to their level of readiness for change [7]. It is known that tailored interventions are more effective for changing health behaviors [11, 12].

There has not yet been a systematic review of adolescent nutritional interventions that adopted the TTM as a theoretical model in the design of the intervention. Moreover, this model consists of many constructs, so it is applied in different ways by different authors. Therefore, this review aimed to weigh the strength of evidence about the TTM usage in nutritional interventions for adolescents, by describing how the TTM (any construct mentioned above or all) is applied to nutritional interventions for adolescents and evaluate its effectiveness regarding dietary intake.

## Methods

This study followed the PRISMA guidelines [13] and is registered on the PROSPERO Website (#CRD42018096819).

### Data sources and search strategy

**Fig. 1:**
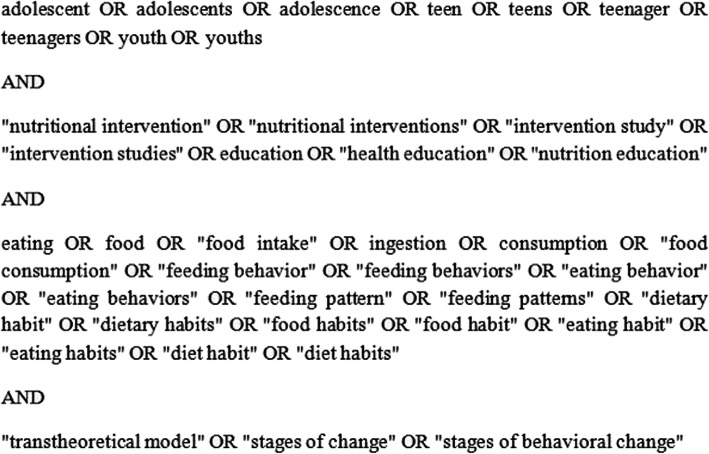
PubMed search strategy

The following databases were searched for articles in English, Portuguese, or Spanish: Adolec, Google Scholar, LILACS, PsycINFO, PubMed, Science Direct and Web of Science. The search was updated twice, once after thirty days and again about a year later to find recently published articles. A librarian from the University of Brasilia assessed the quality of the primary search by filling out the Peer Review of Electronic Search Strategy form. Figure 1 describes the primary search strategy for PubMed, which was then adapted for the other databases. MeSH Terms were applied, such as “adolescent”, “food intake”, and “health education”. Since transtheoretical model was not a MeSH term, and because the stages of change are described differently in nutritional intervention studies, the following free text words were searched: “transtheoretical model”, “stages of change” and “stages of behavioral change”.

### Eligibility criteria

Only randomized controlled trials and quasi-experimental studies that included adolescents between ten and nineteen years who had been exposed to any type of nutritional intervention that used at least one TTM construct in the design were eligible. When the original article only mentioned the use of self-efficacy, it was reviewed if the TTM has been used in the design of the intervention or if another construct of the model has been applied. This criterion was adopted since self-efficacy is a construct of other behavioral change theories as well. No other restrictions were applied, including publication date. Studies involving adolescents were included even if they have included children or young adults. The exclusion criteria were interventions that targeted specific health conditions or diseases, such as type 1 diabetes, cancer, and eating disorders, studies that only classified participants according to stages of change as a variable (i.e., without implementing the TTM as a theoretical basis in the design of the intervention), and studies in which the intervention had not yet been implemented. Studies that targeted individuals with obesity were not excluded.

### Selection process

**Fig. 2:**
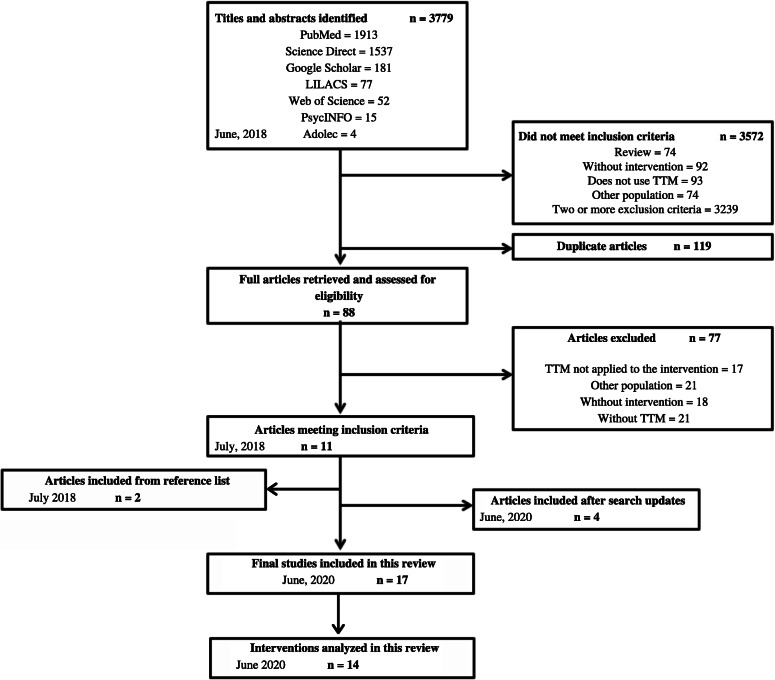
Selection process

A total of 3779 titles and abstracts found in the database search were input into Mendeley software. Duplicates were removed and assessed manually by the first reviewer. Two reviewers performed the study analysis, which included reading the titles and abstracts and discarding those that did not match the inclusion criteria (JN, GRM). Each full paper was read separately by both reviewers (JN, GRM). Disagreements between reviewers were resolved by an expert (NT). One reviewer assessed the reference lists of the included studies to find other related articles (JN). An updated search was performed in the databases by one reviewer (JN), and both reviewers assessed the eligible studies for inclusion (JN, GRM). Figure 2 shows the entire selection process, including dates.

### Data extraction and study quality assessment

The data were extracted using a table based on the Centre for Reviews and Dissemination for Undertaking Reviews in Healthcare instructions [14]. Data were extracted on the publication type, country, funding, main purpose, study design, intervention characteristics (time, frequency of exposure, inclusion/exclusion criteria, methods of delivery) and outcomes (follow-up, dropout rate, and main results), population characteristics (age, gender, ethnicity), and TTM construct measures. Further information about data extraction is available in the supplementary material. Two reviewers performed this process separately, assessing the quality and risk of bias in each study using the Effective Public Health Practice Project Quality Assessment Tool for Quantitative Studies [15]. This questionnaire was designed primarily to assess the quality of interventional studies designed for public health purposes, which is the case of the included studies. According to Olivo et al., the questionnaire provides “excellent agreement for the final grade” of included studies [16]. It extracts information on selection bias, study design, blinding, data collection methods, withdrawals and dropouts, intervention integrity, and analyses performed [16]. The articles were rated for each component and a final global categorical rating was assigned (strong, moderate or weak), as recommended by the Effective Public Health Practice Project [15].

## Results

The initial search returned 3779 results, from which 119 duplicates were removed. Of the remaining abstracts, 3572 did not meet the selection criteria and were excluded. Thus, the full texts of 88 articles were read. Of these, 77 did not meet the eligibility criteria for reasons described in Fig. 2. The final sample of 11 articles was published between 2003 and 2018 [17–29]. Two of the articles were assessed jointly because they covered the same intervention [19, 20]. Two articles on interventions that had already been covered in the review were included through a reference list search. Both of these articles were analyzed with the other studies on the same intervention [18, 27]. After the updated search, four more papers were included [30–33], totaling 17 articles and 14 interventions. All but two of the studies received external funding [31, 32], but it has not represented a potential conflict of interest in any of them.

### Methodological quality of the studies

Five interventions were classified as weak [21, 25, 26, 30, 33], six as moderate [22–24, 29, 31, 32] and three as strong [17, 19, 28]. Most of the studies were rated as weak due to having a non-representative sample [21, 25, 26, 30] and because blinding was not mentioned in the paper [25, 26]. Further information about the articles’ scores and classifications is available in the supplementary material.

### Sampling and recruitment

One study recruited adolescents from youth service agencies [19], another recruited adolescents in search of nutritional counselling [31] and a third recruited adolescents through digital media, radio, and television [30]. The remaining studies recruited adolescents from schools [17, 21–26, 28, 29, 32, 33]. The participants’ age ranged from 7 to 19 years old, with two studies including preteens [29, 32]. The majority of the studies targeted low-income populations [19, 21–23, 25, 28, 31], with three focusing on middle-income adolescents [17, 24, 26], one on African Americans [19] and two exclusively on girls [23, 24]. Four studies focused on adolescents with obesity [24, 29–31].

### Study design characteristics

The sample sizes varied from 16 to 2983 participants. A total of eight randomized controlled trials [17, 23, 25, 26, 28–30, 33] and six quasi-experimental studies [19, 21, 22, 24, 31, 32] were included. All but six of the studies had at least two follow-up measurements [17, 26, 29, 31–33]. The study duration (including pre-test and post-test measurements) ranged from one month to three years, with many lasting six months or more [17, 23–26, 28, 29, 31].

### Intervention strategies and measured variables

The majority of the interventions involved digital technology [17, 19, 21, 23, 25, 26, 28]. Their strategies included the use of websites [17, 26], videos [21] and CD-ROMs [19, 23] as means of providing information and assessment. One intervention involved SMS (Short Messages Service) messages [25]. Two interventions mailed printed materials, such as magazines, letters to families and newsletters [28, 32]. Most of the interventions occurred at school facilities [17, 21–24, 26, 28, 32, 33]. Some studies involved dietary assessment methods, such as food frequency questionnaires, 3-day food records, self-reported consumption, and food diary [19, 21, 22, 28, 33]. Only one study assessed previous nutritional guidance [28]. Excluding outcomes related to dietary intake, some studies also analyzed anthropometric measures (height, weight, Body Mass Index percentiles, waist circumference, waist-to-hip ratio and body fat) [25, 30, 31, 33] and nutritional knowledge [24]. Other characteristics of the intervention can be found in Table 1.

**Table 1:** Main results of the included studies

***Study and Purpose***	***Participants’ Characteristics***	***Intervention***	***Duration of exposure, follow-up, and frequency***	***Main Results***
Boff et al. [30] (2018) To evaluate the effectiveness of a TTM-based intervention on anthropometric, metabolic, and motivational outcomes in adolescents with obesity.	**Sample:** 65 **Age:** 15–18 y **Gender:** Male: 57% Female: 43% **Country:** Brazil	**Who delivered:** Nutritionists, psychologists, and other health professionals. **To whom:** Adolescents who were with overweight or obesity **Format:** Motivational Interdisciplinary Group (IG) and the Traditional Health Education Group (CG) **Context:** Online **Content:** For the IG, the sessions focused on motivation to change eating habits through the stages of change, the processes of behavioral change, and enhanced decision-making and self-efficacy. The CG received traditional education in health. The primary outcomes were changes in TTM variables and anthropometric measures.	**Duration:** 3 months **Follow-up:** Baseline and after 12 weeks **Frequency:** 12 weekly meetings for 1h30min	**Outcome analyzed:** Dietary intake **TTM constructs used:** Processes of change Stages of change Decisional balance Self-efficacy **Main results:** There was a statistically significant difference only in decisional balance between groups over time. No significant differences for dietary intake were found.
Brick et al. [17, 18] (2017) To evaluate stage progression in a large computer-based, TTM- tailored intervention involving physical activity, fruit and vegetable consumption, TV viewing, and substance abuse prevention.	**Sample:** 2983 **Age:** 10–15 y (mean 11.4 y) **Gender:** Male: 52.2% Female: 47.8% **Country:** United States	**Who delivered:** Research assistants **To whom:** Students **Format:** Energy balance intervention and alternate intervention groups **Context:** The intervention was delivered in school computer laboratories using laptops provided by the study **Content:** The energy balance group received an intervention to increase fruit and vegetable consumption. The alternate group received an intervention to prevent/cessate smoking and alcohol use. Both groups received TTM-tailored intervention, and feedback	**Duration:** 3 years **Follow-up:** Baseline, follow up assessment every year for 3 years **Frequency:** 5 sessions	**Outcome analyzed:** Dietary intake **TTM constructs used:** Stages of change **Main Results:** Regarding fruit and vegetable intake, the energy balance group had greater percentages of consumption than the substance use prevention group, progressing to the action or maintenance at 12, 24, and 36 months.
Di Noia et al. [19, 20] (2008) To examine the efficacy of a TTM-based computer-mediated intervention to increase fruit and vegetable consumption among economically disadvantaged African American adolescents.	**Sample:** 507 **Age:** 11–14 y (mean 12.4 y) **Gender:** Male: 39% Female: 61% **Country:** United States	**Who delivered:** Research staff **To whom:** African American adolescents from Youth services agencies **Format:** Computer intervention (CIN) and Control **Context:** CD-ROM mediated intervention content in Youth services agencies **Content:** The intervention addressed the health benefits of consuming five or more daily servings of fruits and vegetables. The CIN received stage-tailored sessions	**Duration:** 4 weeks **Follow-up:** 2 weeks before and after the intervention **Frequency:** 4 onsite 30-min weekly sessions	**Outcome analyzed:** Dietary intake **TTM constructs used:** Processes of change Stages of change Decisional balance Self-efficacy **Main Results:** The fruit and vegetable intake of those involved in the program increased about 38% more than the control group, an average increase of 0.9 daily servings of fruits and vegetables. More youths in the intervention than in the control group progressed to later stages.
Filgueiras et al. [31] (2018) A multidisciplinary TTM-based motivational intervention involving nutritional counseling for low-income adolescents with obesity.	**Sample:** 16 **Age:** 11–17 y **Gender:** Male: 57% Female: 43% **Country:** Brazil	**Who delivered:** Nutritionists and psychologists **To whom:** Adolescents with obesity **Format:** Individual nutritional counseling and nutritional education workshops **Context:** The nutritional education workshops were conducted in the Center of Nutritional Recovery and Education (CREN) **Content:** All participants went through individual nutritional counseling, according to their stage, in a CREN office, to help them overcome the difficulties and barriers involved in changing dietary habits, reinforcing the positive aspects of the changes that had already been made.	**Duration:** 13 months **Follow-up:** Baseline, 6 and 13 months **Frequency:** Once a week	**Outcome analyzed:** Dietary intake **TTM constructs used:** Stages of change **Main Results:** At the beginning, about 70% of the participants were in the precontemplation stage. After six months, 60% had changed to the action stage. At the end of the intervention, all participants had reached the action or maintenance stages.
Freen et al. [21] (2005) To examine the effectiveness of 8 sessions of a TTM/Health promotion intervention (Internet/video-based) to increase physical activity and reduce dietary fat among 7th graders.	**Sample:** 103 **Age:** 12–14 y **Gender:** Male: 40.6% Female: 59.4% **Country:** United States	**Who delivered:** Research staff **To whom:** Students **Format:** Control group and Intervention group **Context:** The intervention was conducted in a computer laboratory where each student had a computer **Content:** The focus of the intervention was on reducing dietary fat with strategies appropriate for all stages of change, particularly for those in precontemplation and contemplation stages	**Duration:** 1 month **Follow-up:** 1 week before and after intervention **Frequency:** 8 sessions of 40 min (1 class period)	**Outcome analyzed:** Dietary intake **TTM constructs used:** Processes of change Stages of change **Main Results:** Among those who participated in more than half the sessions, dietary fat decreased from 30.7 to 29.9% of the total calorie intake. The diet of those who participated in less than half of the sessions was not significantly different than the control group.
Freen et al. [22] (2003) A stage-based intervention to reduce fat consumption in middle school students.	**Sample:** 74 **Age:** 12–17 y (mean 13.82 y) **Gender:** Male: 47% Female: 52% **Country:** United States	**Who delivered:** Graduate nursing students in pediatric nursing **To whom:** Students **Format:** Control group and Stages of change intervention group **Context:** All classroom interventions took place during the Family and Consumer Education class **Content:** Classroom interventions incorporated processes appropriate for the precontemplation and contemplation stages of change by using multiple instructional methods appropriate to middle school students, content to increase knowledge, and peer modeling of skills	**Duration:** 4 class periods **Follow-up:** Pre-test,and post-test **Frequency:** 4 sessions of 45 min	**Outcome analyzed:** Dietary intake **TTM constructs used:** Processes of change Stages of change Decisional balance Self-efficacy **Main Results:** The average percentage of fat in dietary intake ranged from 30.7 to 32.8%; the percentage of fat increased less in the intervention group than the control group.
Gur et al. [32] (2019) To evaluate the impact of a Transtheoretical Model-based programme titled ‘Fruit & Vegetable-Friendly’ on the fruit and vegetable (F&V) consumption of adolescents.	**Sample:** 702 **Age:** 9–15 y (mean 12.02 y) **Gender:** Male: 45.2% Female: 54.8% **Country:** Turkey	**Who delivered:** Research team **To whom:** Students and their families **Format:** Single group **Context:** The intervention took place in the classroom **Content:** The intervention presented different components in order to address every stage.	**Duration:** 8 weeks **Follow-up:** Baseline, post-intervention, and 6 months after the intervention. **Frequency:** Not reported	**Outcome analyzed:** Dietary intake **TTM constructs used:** Processes of change Stages of change Decisional balance Self-efficacy **Main Results:** The difference in consumption of fruit and vegetable six months after the intervention was 3·7 portions/d for those who were in the precontemplation stage, 3·0 portions/d in those in the contemplation stage and 2·4 portions/d in those in the preparation stage. The difference for those in the action stage was 0·8 portions/d. In the maintenance stage, total F&V consumption had decreased by 1·2 portions/d. Students in the action and maintenance stages increased, while the percentage of students in the precontemplation, contemplation and preparation stages decreased.
Haerens et al. [23] (2007) To examine the mediating effects of changes in psychosocial determinants of dietary fat intake on changes in fat intake in adolescent girls.	**Sample:** 788 **Age:** 11–15 y (mean 12.9 y) **Gender:** Female: 100% **Country:** Belgium	**Who delivered:** School staff **To whom:** Female students **Format:** Intervention and Control groups **Context:** The intervention occurred during class hours **Content:** The students completed a youth-based version of the computer-tailored fat intake intervention. The TTM was used to define the content and approach of feedback.	**Duration:** 1 h **Follow-up:** Baseline and 1 year after intervention **Frequency:** 1 class hour	**Outcome analyzed:** Dietary intake **TTM constructs used:** Self-efficacy Decisional balance **Main Results:** On average, fat intake in the intervention group was reduced by 9.0 g/day vs. the control group.
Jalambadani et al. [24] (2017) To investigate the effects of education (TTM) on reducing fast food consumption among female adolescents suffering from obesity and overweight in Sabzevar, Iran.	**Sample:** 420 **Age:** 15–18 y (mean 16.36 y) **Gender:** Female: 100% **Country:** Iran	**Who delivered:** Research staff **To whom:** Female students with obesity **Format:** Education and Control groups **Context:** The intervention took place in the classroom **Content:** The education group participated in meetings that focused on nutrition concepts and identified methods to stay motivated. The meetings also included discussion with students on difficulty and ease in consumption reduction of fast food.	**Duration:** 12 weeks **Follow-up:** Pre-test, and post-test **Frequency:** 60 min, twice a week	**Outcome analyzed:** Dietary intake and nutritional knowledge **TTM constructs used:** Processes of change Stages of change Decisional balance Self-efficacy **Main Results:** The average rates of stages of change, processes of change, and self-efficacy in the education group improved significantly. No statistical significance was obtained for decisional balance between the two groups after the intervention. No significant differences for dietary intake were found.
Lana et al. [25] (2013) To assess the impact of a web-based intervention supplemented with text messages to reduce cancer risk linked with smoking, unhealthy diet, alcohol consumption, obesity, sedentary lifestyle and sun exposure.	**Sample:** 737 **Age:** 12–16 y **Gender:** Male: 45.2% Female: 54.8% **Country:** Spain and Mexico	**Who delivered:** Self-delivered **To whom:** Students **Format:** Experimental group 1 (EG1), Experimental group 2 (EG2), and Control group **Context:** Online **Content:** The EG1 and EG2 members had free access to a tailor-made and interactive website. During the academic year, this website was periodically updated with different school and leisure activities related to the avoidance of risk behaviors. The EG2 also received encouraging text messages. Cancer risk behaviors, such as not eating enough fruits and vegetables and being overweight were assessed before and after the study.	**Duration:** 9 months **Follow-up:** Baseline and post-test **Frequency:** 9 months of website access	**Outcome analyzed:** Dietary intake **TTM constructs used:** Stages of change **Main Results:** Both groups decreased by more than 70% the number of students who did not consume enough fruit.
Mauriello et al. [26, 27] (2010) To report on the effectiveness of Health in Motion, a computer tailored multiple behavior intervention for adolescents.	**Sample:** 1800 **Age:** Mean 15.9 y **Gender:** Male: 49.2% Female: 50.8% **Country:** England	**Who delivered:** Research assistants **To whom:** Students **Format:** Multimedia intervention and Control groups **Context:** All sessions were administered via computers in school computer laboratories **Content:** Students self-directed through the 30-min program in which they completed a series of TTM-based assessments and received stage-matched and tailored feedback messages related to fruit and vegetable consumption based on their responses.	**Duration:** 2 months **Follow-up:** Baseline and after 6 and 12 months **Frequency:** 3 sessions	**Outcome analyzed:** Dietary intake **TTM constructs used:** Stages of change **Main Results:** The multimedia intervention group reported eating significantly more servings of fruits and vegetables than the control group at 2 months, 6 months, and 12 months. Individuals within the intervention group were found 1.4–4.2 times more likely to progress to action or maintenance.
Muzaffar et al. [33] (2019) To evaluate the afterschool PAWS (Peer-education About Weight Steadiness) Club program delivered by peer or adult educators to improve food choices, physical activity, and psychosocial variables related to healthy eating.	**Sample:** 109 **Age:** 11-14y **Gender:** Male: 30% Female: 70% **Country:** United States	**Who delivered:** Educators **To whom:** Students **Format:** Peer-led and adult-led groups **Context:** The intervention occurred at school **Content:** The curriculum was focused on building healthy eating patterns and addressing stages of change variables. Printed goal-setting worksheets were provided to the participants at each of the 12 sessions.	**Duration:** 12 weeks **Follow-up:** Baseline, post-intervention, and 6 months after the intervention **Frequency:** Weekly sessions of 1h30min	**Outcome analyzed:** Dietary intake **TTM constructs used:** Stages of change **Main Results:** All participants significantly reduced kcals/day from baseline to 6-months post-intervention. For the peer-led group, self-reported intake of whole grains (servings/day) increased from baseline to 6-months post-intervention.
Toral et al. [28] (2012) To assess the impact of a six-month stage-based intervention on fruit and vegetable intake for perceived benefits, barriers, and self-efficacy among adolescents.	**Sample:** 771 **Age:** 11–19 y **Gender:** Male: 40.5% Female: 59.5% **Country:** Brazil	**Who delivered:** Research staff **To whom:** Students **Format:** Intervention Group and Control Group **Context:** The materials were distributed in classrooms and by mail **Content:** The students received printed educational materials for promoting healthy dietary habits, both in classrooms and by mail. The materials were directed toward the participants’ stages of change.	**Duration:** 6 months **Follow-up:** Baseline, and follow-up assessment after the intervention **Frequency:** 6 monthly newsletters and magazines	**Outcome analyzed:** Dietary intake **TTM constructs used:** Processes of change Stages of change Decisional balance Self-efficacy **Main Results:** No significant changes were found in fruit and vegetable intake, benefits, barriers, or perceived self-efficacy.
Yusop et al. [29] (2018) To assess the effectiveness of a stage-based lifestyle modification intervention for children with obesity.	**Sample:** 40 **Age:** 7–11 y (mean 9.8 y) **Gender:** Male: 52.5% Female: 47.5% **Country:** Malaysia	**Who delivered:** Dietitians and physical education professionals **To whom:** Students with obesity and parents **Format:** Intervention group and Control group **Context:** The intervention study was conducted at an university Dietetic Clinic **Content:** Intervention group received stage-based lifestyle modification intervention based on the Nutrition Practice Guideline for the Management of Childhood Obesity, while control group received standard treatment.	**Duration:** 24 weeks **Follow-up:** Baseline, follow up every month and at the end of the intervention **Frequency:** 3 sessions of 2 h of aerobic exercise on weekends (once every 2 months); 1 h of Nutritional counseling every week.	**Outcome analyzed:** Dietary intake **TTM constructs used:** Stages of change **Main Results:** Dietary intake was not significantly different between the two groups.

### Nutritional topic covered in the intervention

Six aimed at improving fruit and vegetable consumption [17, 19, 25, 26, 28, 32], three promoted a low-fat diet [21–23], three focused on healthy dietary intake in general [29–31], one focused in improving food choices and cooking skills [33], and one focused on the reduction of fast food intake [24].

### Application of the TTM

All studies but one used the stages of change in the development of the intervention [23]. The majority of them used the stages of change to create a tailored intervention, with the exception of one study that used the stages to direct the content of the intervention to all five stages of change progressively [33]. Six studies exclusively used the stages of change’ construct [17, 25, 26, 29, 31, 33]. Seven used the processes of change in accordance with the stages of change to create a tailored intervention [19, 21, 22, 24, 28, 30, 32]. Five studies included decisional balance and self-efficacy as a measure [19, 24, 28, 30, 32] and one used these two constructs in the development of the intervention [23].

Boff et al. scheduled 12 meetings conducted by professionals in different areas, such as psychology, nutrition, physical therapy, and physical education. The meetings were based on the stage of the intervention group [30]. Brick et al. provided tailored TTM-based computer intervention sessions for three groups according to their grade [17]. The TTM-based intervention program of Di Noia et al. included an introductory session and a stage of change assessment, which was followed by three stage-based sessions involving the most suitable change strategies. For those in the precontemplation stage, consciousness raising, dramatic relief and environmental reevaluation were used. For the contemplation/preparation sessions, self-reevaluation and self-liberation were incorporated. For the action/maintenance stages, change reinforcement management, helping relationships, counterconditioning, and stimulus control were used [19]. Filgueiras et al. provided nutritional counseling that set specific stage-based behavioral change goals, as well as nutritional education workshops [31]. The intervention in Frenn et al. was designed for the whole class, focusing on processes of change appropriate for those only in the precontemplation and contemplation stages. Individual stage-based computer-generated feedback on dietary fat was provided. The processes of change used for the whole class were self-reevaluation and consciousness raising. Decisional balance was explored in half of the intervention sessions, whose topics were reducing barriers to healthy foods and emphasizing its benefits [21]. Frenn et al. provided four class sessions based on processes of change, consciousness raising and self-reevaluation, because the majority of their participants were in the precontemplation or contemplation stages. Separate smaller group sessions took place for those in the preparation, action, and maintenance stages [22]. Intervention from Gur et al. presented different components in order to address every stage. Examples of the strategies included a card game to promote the pros of eating F&V and explain their various features [32]. Haerens et al. used concepts of self-efficacy and the benefits and barriers to define the content of and feedback about a fat consumption intervention [23]. Jalambadani et al. provided lessons on identifying and overcoming barriers related to the reduction of fast food consumption and methods for staying motivated. The curriculum also included information on processes of change and self-efficacy [24]. The participants in Lana et al. accessed a website based on attitude, social influence, and self-efficacy theory and TTM, sending SMS messages to increase self-efficacy [25]. In the study by Mauriello et al., the intervention group received stage-matched, tailored feedback messages based on their TTM assessments, which included all TTM constructs [26]. The non-tailored intervention of Muzaffar et al. provided 12 weekly meetings that included small group discussions led by the educators, hands-on and food preparation activities, and facilitated group decision-making and problem-solving experiences for participants. All content was developed based on all stages of change of the TTM [33]. Toral et al. developed and mailed printed educational materials promoting healthy dietary habits according to their stage of change to the intervention group [28]. Yusop et al. included nutritional counseling in an intervention that was tailored to the participants’ current stage of change. The nutritional counseling topic was based on the participant’s current stage of change [29].

### Other theoretical bases

The TTM was used in all included interventions, although some of them were based on other theories as well. Two studies used a combination of Health Promotion and TTM [21, 22], one used determinants from social cognitive theory [33], another study used a combination of the social cognitive theory and the theory of planned behavior [23], and one article used the Attitudes Social influence Self-efficacy model [25].

### Intervention duration and frequency of exposure

In one study the participants were exposed to the intervention only once [23], while five interventions had weekly sessions [19, 24, 29–33], and another sent magazines and newsletters once a month [28]. One study enabled access to a website for nine months, including teacher support [25].

### Main outcomes

#### Fruit and vegetable intake

There was an improvement in fruit and vegetable consumption in the intervention groups of four studies [19, 25, 26, 32] and four also progressed through stages of change [17, 25, 26, 32]. Participants of intervention of Di Noia et al. increased fruit and vegetable consumption more than controls. Besides, more participants of the intervention group progressed to later stages and maintained recommended intake levels [19]. In the intervention from Gur et al., students in the action and maintenance stages increased, while the students in the other stages decreased [32]. In the study of Lana et al., the number of students who did not consume enough fruit decreased by more than 70% [25]. The intervention group of Mauriello et al. reported eating significantly more servings of fruits and vegetables than the control group [26]. Toral et al. found no differences in the participants’ fruit and vegetable intake or in perceived self-efficacy or benefits and barriers compared to baseline [28].

#### Dietary fat intake

There were positive results in intervention groups of Haerens et al. and Freen et al. regarding fat intake reduction [21, 23]. Nevertheless, the percentage of fat increased in Freen et al.’s intervention group, although it was significantly less than the control group [22].

#### Other outcomes

All the participants in Filgueiras et al. were in the action or maintenance stages by the end of the intervention [31]. In the intervention of Muzaffar et al., all participants significantly reduced kcals/day from baseline to 6-months post-intervention. For the peer-led group, self-reported intake of whole grains (servings/day) increased from baseline to 6-months post-intervention [33]. In Boff et al., Jalambadani et al., and Yusop et al. study, no significant differences between groups for dietary intake were found [24, 29, 30].

## Discussion

This is the first systematic review to collect data on how the TTM has been applied to design nutritional interventions for adolescents. This review also showed the effectiveness of each intervention. Although five studies were considered weak in the quality assessment, one of the reasons for it is the fact that the tool for assessment takes into account the blinding, which is hardly feasible in studies with this type of intervention. The majority of studies used the TTM, most specifically the stages of change, to develop a tailored intervention program. According to Celis-Moralez et al., tailored interventions that incorporate behavior change techniques are more effective than conventional interventions for dietary behavior change [12]. This shows the TTM is a well-known method of tailored interventions [34].

At all times the processes of change were used in combination with the stages of change and also with the aim of tailoring the content of the intervention, except for Jalambadani et al. [24], which included process of change as a variable. In addition, this construct was not found to be used exclusively. According to Velicer et al., using the TTM in interventions can result in higher retention rates, because the participants’ motivation level is adequate for the objectives of the intervention program [35]. One recent intervention [33] used the stages of change to gradually organize the content of the intervention, ensuring that individuals of all stages could receive at least one content matched to their corresponding stage. This study found positive results in the intervention and opens the way for an alternative to a tailored intervention, demanding less time and logistics.

This review points out the fact that decisional balance and self-efficacy are mostly used as a measure of outcome, rather than a tool for the development of interventions. Only one study [23] used these constructs to define the content of and feedback of a nutritional intervention, showing that most studies that claim to be based on theoretical models use, in fact, a part of the model in the design of the intervention, which increases the possibility of variability of results from one intervention to another and decreases the possibility of replication. This statement can be confirmed by the fact that no study used all constructs in the development of the intervention, and only six used all four constructs of the TTM, even as a measure of outcome. Finally, the stages of change show to be the most well-known construct of the TTM, since all interventions, but one, used it, and, of these, six studies used exclusively this construct.

Using the constructs as a measure shows to be positive for interventions. The literature shows TTM as a more sensitive measure of progress for a dietary intake change, and even when the food consumption is not altered, an increase in decisional balance or self-efficacy, or progress through the stages of change can represent a positive outcome [35].

Many studies recruited adolescents from schools, a normal setting for health programs [36], and nine of them also implemented the intervention at the school’s facility [17, 21–24, 26, 28, 32, 33]. Implementing healthy eating programs at schools is recommended by the World Health Organization [37]. In addition, school health programs tend to be more cost-effective [37], a finding also found in this review, since only one of these mentioned above did not obtain positive significant results [28]..

All the studies focusing on low income populations had significant results regarding dietary intake, such as increased consumption of fruits and vegetables, decreased consumption of fat and progress between stages [19–23, 31]. Targeting this group is extremely important because they are more likely to develop health problems, and these odds can be reduced by changing dietary habits [38].

Two interventions focused only on girls, and both had positive results [23, 24]. A study exploring food preferences by gender and age found some differences: boys preferred more meat and fish, and girls preferred more vegetables and sweets [39]. Besides, girls tend to be more concerned than boys about weight loss, engaging in dieting, and present more guilt over eating too much [40]. These findings suggest that gender preferences should be considered when developing nutritional interventions. When the topic is specifically tailored to the study population in terms of stages of change, gender and other characteristics, the intervention tends to become more effective [34, 41].

Guerra et al. found that interventions with longer durations have more positive results [42]. According to the TTM, at least six months are required to maintain a behavior. Few studies continued six months or more [17, 25, 28, 29, 31], so the time period is a positive aspect of their design [42]. On the other hand, there were only two follow up assessments in most of the studies, which is a weakness [19, 21–25, 28, 30]. Moreover, it seems the interventions using TTM focusing at a single dietary intake component, such as consumption of fruit and vegetable or fat intake, showed better results, which has already been concluded in a previous systematic review [11] and is expected to occur, considering the model was initially created to alter a single health behavior (Tobacco use) [7].

Some of the studies used a combination of the TTM and other behavioral change theories. To provide a more detailed explanation of health behavior and to reduce complexity, researchers have been trying to integrate diverse theories [43]. Combining theories is useful when their constructs are complementary, since their strengths can be kept and their weaknesses removed, thus a broader range of factors can be explored [44]. For instance, social cognitive theory analyzes the social effects on behavior in a more representative way than TTM, so if these models are combined, gaps are filled in the latter. In a recent meta-analysis, Gourlan et al. found that physical activity interventions based on a single theory presented better outcomes than interventions based on a combination of theories [45]. However, it is unclear whether this conclusion is also valid for nutritional interventions.

This review reinforced the flexibility of the model, since it was applied in different contexts and in a variety of ways: the interventions occurred at schools, dietetic clinics, youth service agencies and online, and the strategies included printed materials, nutritional counseling, group meetings, classroom lessons and digital technology. Many studies used information and communication technologies in their interventions, including strategies involving websites, videos, and CD-ROMs. This is a positive point because these media are highly acceptable among adolescents. Moreover, using digital media facilitates stage-based tailored interventions [11].

Although the effectiveness of the TTM was addressed in the included studies, they had significant methodological differences, such as the outcome of interest, setting, forms of delivering the intervention, and other characteristics. No studies compared the effectiveness of different theoretical models for changing dietary intake or even if it is better not to use them [46]. In addition, a wide range of factors makes it difficult to determine whether behavior-change techniques are actually effective: many studies used a combination of two or more theories and effectiveness depends on the way a theory is used, as well as on the study population and design [11, 47]. Moreover, the studies do not report the use of behavior change techniques in a precise way, such as the behavior change technique taxonomy proposed by Michie et al. [47].

This review showed that most authors use mainly stages of change when developing nutritional interventions, although a study has shown that it is possible to use the less explored constructs in the design of the intervention [23]. These interventions are presented as TTM based, although according to Mastellos et al. [48] it is better to categorize these interventions as being based on stages of change construct, as three of four constructs are not being taken into consideration. Only when interventions in this field consider the usage of all constructs of TTM for the development, the literature will reach sufficient evidence about the use of the model. At the present moment, the evidence is linked to the usage of the stages of change as a way of tailoring the intervention, which cannot be expanded to the use of TTM, since there are other ways to tailor an intervention.

A meta-analysis could not be performed due to the heterogeneity of the studies. Non-representative samples, low frequency, and exposure to the content of the intervention, and short length of follow-up assessment, that were aspects related to the poor quality of the articles included, were the main limitations of the selected studies. A limitation of this review is the fact this present study was aimed at evaluating effects only on dietary intake. However, an individual’s eating behavior involves several determinants, which were not considered in this review.

## Conclusion

The TTM seems to be a successful strategy for nutritional intervention aiming at improving dietary intake in adolescents. Besides, its application in different contexts shows that the TTM is flexible and possible to be completely implemented in many settings. It is expected that interventions using TTM focusing on a single dietary intake component show better results. The use of the model in the development of the intervention is shown to be restricted to the stage of change’ construct. Although the effectiveness of the TTM was addressed in the included studies, they had significant methodological differences, such as the outcome of interest, setting, forms of delivering the intervention, and other characteristics. No studies compared the effectiveness of different theoretical models for changing dietary intake or even if it is better not to use them [46]. In addition, a wide range of factors makes it difficult to determine whether behavior-change techniques are actually effective: many studies used a combination of two or more theories and effectiveness depends on the way a theory is used, as well as on the study population and design [11, 47]. Moreover, the studies do not report the use of behavior change techniques in a precise way, such as the behavior change technique taxonomy proposed by Michie et al. [47].

This review showed that most authors use mainly stages of change when developing nutritional interventions, although a study has shown that it is possible to use the less explored constructs in the design of the intervention [23]. These interventions are presented as TTM based, although according to Mastellos et al. [48] it is better to categorize these interventions as being based on stages of change construct, as three of four constructs are not being taken into consideration. Only when interventions in this field consider the usage of all constructs of TTM for the development, the literature will reach sufficient evidence about the use of the model. At the present moment, the evidence is linked to the usage of the stages of change as a way of tailoring the intervention, which cannot be expanded to the use of TTM, since there are other ways to tailor an intervention.

## Supplementary information

**Additional file 1.**

**Additional file 2.**

## Data Availability

All data generated in this study is available in supplementary materials.

## Abbreviations

TTM: Transtheoretical Model
PRISMA: Preferred Reporting Items for Systematic reviews and Meta-Analyses
